# Assessment of psychosocial factors in office and operational groups of employees of a Regional Electricity Distribution Company in Iran – A case study

**DOI:** 10.1016/j.heliyon.2018.e00714

**Published:** 2018-08-06

**Authors:** Omid Aminian, Ataollah Moradi, Sahar Eftekhari

**Affiliations:** Center for Research on Occupational Diseases, Tehran University of Medical Sciences, Tehran, Iran

**Keywords:** Psychology, Psychiatry

## Abstract

**Background:**

Many studies have shown an association between unfavorable psychosocial factors and personal and organizational outcomes. In recent years, psychosocial issues have received top priority between work environment factors. This study aimed to provide a first insight into psychosocial stressors in electricity distribution industry in Iran and compare the psychosocial factors between two job categories of office and operational workers.

**Methods:**

The study population was employees of an electricity distribution company in Iran. The standard Persian medium size of Copenhagen psychosocial questionnaire (COPSOQ) was used in this study to measure psychosocial factors. The questionnaire comprised 5 domains and 26 scale. Mean and standard deviation of each scale and domain were obtained for office and operational workers separately and the results were compared.

**Results:**

Higher scores were obtained in the domain of "job demands" of operational workers. In the domain of "job contents", More Unfavorable condition was obtained for office workers. No significant difference was seen in the domain of "inter personal relationship". More unfavorable scores for operational workers were obtained in the domain of work-individual interface" in a univariate analysis, but after adjusting the confounders including age, type of employment, directorship status and income, multivariate analysis did not show any significant difference. In the domain of "individual Health and well-being", higher scores were obtained for operational workers.

**Conclusion:**

This study provides support regarding the differences of psychosocial working environments between office and operational workers in electrical distribution industry. It is worth considering these differences of psychosocial factors at employees scheduling.

## 1 Introduction

The World Health Organization defines health as complete physical, mental and social well-being rather than merely absence of disease or infirmity [1]. A healthy workplace is one in which there is an absence of harmful conditions to health, moreover the combined efforts of employers, employees and society should be made toward health promotion of the workers. Work-related stress occurs when people are challenged by work pressures and requirements that are incompatible with their knowledge or ability [2].

Occupational stress is one of the most important health risks for employees. According to studies in Europe, one out of three working people suffers from occupational stress. Studies have shown that 50%–60% of the lost working days were caused by occupational stress [3, 4, 5, 6].

Several studies have shown the association between adverse psychosocial factors and various outcomes including personal consequences like diabetes [7], musculoskeletal disorders [8], work-family conflict [9], adverse lifestyle [10], and mental health [11] as well as organizational adverse effects such as absenteeism [12], intent to leave the job [13], also, in some studies, an association is found between occupational stress and occupational accidents [14, 15].

Although occupational stress is not limited to the developed world and there is a growing concern in developing countries, there still is inadequate national or regional information in this regard and many of work related OSHA policies and programs, partially in low and middle income countries, concentrated on traditional workplace exposures [16, 17].

To assess workplace psychosocial factors, subjective data obtained from interviews or questionnaires should be used. In recent years, the Copenhagen Psychosocial Questionnaire (COPSOQ), developed by Tage S. Kristensen and Vilhelm Borg at the National Institute of Occupational Health in Copenhagen-Denmark, has commonly been used. This is a theory-based questionnaire that is not limited to a single theory, and is a relatively novel and comprehensive questionnaire consisting main dimensions of workplace psychosocial factors. This instrument is useful for international comparisons and not only measures the defined potentially health hazards at work as other questionnaires, but assesses all aspects of psychosocial work environment [18, 19, 20, 21].

The power industry is involved in electricity generation, transmission, and distribution processes. There are several stressors including physical, chemical, ergonomic, and psychological factors in this industry [22]. Traditionally, there was a great interest in assessing adverse outcomes caused by physical and chemical work hazards particular in blue-collar workers, but due to globalization in recent years, various changes in work activities and organizations resulted in increasing level of psychosocial hazards [23]. The indirect impact of occupational stress on organizational productivity has been demonstrated through the effect of job satisfaction on the productivity of the employees [24]. Moreover, among the electricity company's employees, occupational stress has been significantly associated with health disorders [25, 26], and the prevalence of depression and common mental disorders has been associated with adverse psychosocial factors [27, 28]. In the context of task, maintenance of electrical transmission has potential psychosocial risks such as living in danger and being responsible for well function of the electricity system [29]. Therefore assessing psychosocial work environment with a comprehensive tool can be helpful for organizational scheduling toward work condition promotion.

There are two groups of employees including office and operational employees in electricity distribution companies; the former mainly work in the office environment, and deal with subscriptions, billing, design of distribution networks, and statistics, and the latter, mainly perform network repair and maintenance, and commissioning and installation of new distribution networks. We assumed that workers in two different occupational groups facing different psychosocial stressors and a wide-ranged assessment approach is needed to reveal psychosocial workload in two groups. Some previous studies used COPSOQ questionnaire to compare psychosocial factors between white and blue collar workers and concluded that this questionnaire was better suited to assess and compare the variety psychosocial factors at work in different occupational groups [30, 31].

In the present case study, psychosocial factors of one electricity distribution company workers were assessed in multidimensional concepts. As a very few studies have investigated work stress in electricity distribution industry in Iran, findings of current study can provide a first insight and reveal particular psychosocial hazards of electric power industry in Iran. This study aimed to establish a clear understanding about how best to proceed in addressing the existing problems and offer a new set of recommendations for further extended investigations.

## 2 Materials and methods

### 2.1 Study population and data collection

The survey designed as a case study research. Participants consisted of office and operational employees of one of the largest electricity distribution companies in Iran, which is located near Tehran, capital of Iran. The data were collected from August 2014 to February 2015 through paper based questionnaires with oral and writing explanation about purpose of the study. An occupational physician from the project team was present during data collection to provide adequate information and guarantee anonymity and voluntary participation in study. Employees with less than one year's experience were excluded. So that the results can be more probably contributed to the current job of the workers.

### 2.2 Measurements

Workplace psychosocial factors were measured and compared in both occupational groups using the medium size of the first version of COPSOQ [18]. The valid Farsi version of the questionnaire was adopted by Arsalani et al in 2011 [32], which contained 26 scales in 5 main domains and 95 items. With the exception of four 2-option items, the remaining items were in a 5-option format. Five response options either qualified by intensity (from “to a very large extent” to “very small extent”) or frequency (from always to never/hardly ever) were available. [18]. Internal correlation of the scales were verified (Cronbach alpha = 0.84), which shows an acceptable internal validity.

The first part of the questionnaire deals with demographic details (age, sex, marital status and level of education), smoking status, working patterns including occupational group (office or operational), working experience in years, working hours per week, employment type (formal or conventional), being shift worker, having directional relation, and history of job accidents or near misses in the past year (one question).

From a theoretical point of view, COPSOQ questionnaire is not taken into account in the effort-reward imbalance [18]. As financial reward based on the person effort can exert influence on job satisfaction, we asked the workers income per month and included this variable in linear model.

### 2.3 Statistical analysis

The data were analyzed in SPSS-20. All questions were of equal weight, and scores were from 0 to 100. Scores of each scale calculated as mean of scores of the single items, if at least half of the single items had valid answers. Therefore all scales had a theoretical score from 0 to 100. High scores indicated high level of measured concepts and better condition, except in scales relating to demand, role conflict, insecurity at work, and the three scales associated with stress in which high scores indicate higher psychosocial stressors [18]. We computed job demand index, job content index, interpersonal relationship index, work−individual interface index and health and well-being index by summing up scores of relevant scales (after adjusting for the scoring direction). This resulted in scores from 0 to 500 for job demand and job content domains with five scales, 0–800 for interpersonal relationships and leadership domain with eight scales, 0–200 for person−work interface with two scales and 0–600 for the health and well being domain with six scales. A higher score indicates more unfavorable psychosocial conditions in the workplace [18, 33, 34, 35, 36].

The Chi-square test was used to analyze group differences. Two occupational groups were compared in terms of psychosocial factors in 26 scales and 5 domains by univariate analysis using independent samples t-test. The two groups were compared using mean and SD of each scale and domain. We applied p < 0.05 as criterion for statistical significance. Multivariate linear regression analysis was performed to find out if belonging to white or blue collar groups is a potential predictor of the scores of five main domains of COPSOQ questionnaire after controlling for other relevant independent variables.

### 2.4 Ethical considerations

Study objectives were explained verbally and in writing. Participation was on voluntary basis, and the questionnaires remained anonymous. This study was approved by the Medical Ethics Committee of Research Deputy of the Ministry of Health.

## 3 Results

Of the 700 questionnaires distributed, 521 were completed (response rate: 74.4%), of whom, 257 belonged to office and 264 to operational employees. The majority of the participants were male (437 men, and 84 women). Almost all participating women were in the office group (97%). The participants' mean age was 34.02 ± 7.8 years (34.3 ± 8.15 office and 33.8 ± 7.6 operational groups). The mean work experience and hours/week were 9.4 ± 6.9 years (9.88 ± 7.1 office and 8.99 ± 6.63 operational) and 49.1 ± 9 hours (47.49 ± 7.21 office and 50.87 ± 10.31 operational), respectively. Table 1 shows the frequency of other demographic details, job patterns, and smoking. White-collar workers had higher education level comparing to blue-collar workers (P value <0.001). More participants in operational group were shift worker and worked more than 50 hours per week. (P value <0.001 and P value: 0.021 respectively).

**Table 1:** Comparison of demographic and work patterns in two job groups.

	Office workers (N = 257)	Operational workers (N = 264)	P value
	Number (%)	Number (%)	
**Age:**			0.49
≤40	170 (66.1)	174 (65.9)	
>40	87 (33.9)	90 (34.1)	
**Gender:**			<0.001*
Male	175 (68.1)	256 (97)	
Female	82 (31.9)	8 (3)	
**Marital status:**			0.006*
Married	177 (68.9)	210 (79.5)	
Single	80 (31.1)	54 (20.5)	
**Education:**			<0.001*
High school diploma & less	52 (20.2)	153 (58)	
Above diploma	205 (79.8)	111 (42)	
**Type of employment:**			<0.001*
Formal	83 (32.3)	50 (18.9)	
conventional	174 (67.7)	214 (81.1)	
**Working years:**			0.25
≤10	167 (65)	180 (68.2)	
>10	90 (35)	84 (31.8)	
**Working hours per week:**			0.021*
≤50	191 (74.3)	174 (65.9)	
>50	66 (25.7)	90 (34.1)	
**Directorship relation:**			0.51
No	220 (85.6)	231 (87.5)	
Yes	37 (14.4)	33 (12.5)	
**Income per month (million Rials):**			0.67
≤10	81 (31.5)	88 (33.3)	
>10	176 (68.5)	176 (66.6)	
**Smoking:**			0.26
No	232 (90.3)	234 (88.6)	
Yes	25 (9.7)	30 (11.4)	
**Shift working at 1 year ago:**			<0.001*
No	184 (71.6)	102 (38.6)	
Yes	73 (28.4)	162 (61.4)	
**Job accident or near miss in blue collars at 1 year ago:**			0.55
No	254 (98.8)	222 (84.1)	
Yes	3 (1.2)	42 (15.9)	

∗ Statistically significant relationship (p < 0.05).

Univariate analysis showed significant differences between two groups in 4 out of 5 domains, including job demand, job content, work-individual interface, and health (Table 2). The operational group scored higher and reported more stress in job demand and work-individual interface domains. The office workers had higher score and worse state in job content and health domains.

**Table 2:** Comparison of average scores and standard deviations of the COPSOQ scales and domains in two job groups.

Context and level of dimensions	Scales	Office Workers Mean (SD)	Operational Workers Mean (SD)	P value
**D1: Type of production &tasks (Work place)**		268.31 (65.74)	299.39 (66.77)	**<0.001**∗
	1. Quantitative demands	53.37 (18.21)	56.07 (19.72)	**0.10**
	2. Cognitive demands	63.11 (17.35)	71.52 (16.84)	**<0.001**∗
	3. Emotional demands	40.51 (22.46)	41.54 (23.67)	**0.614**
	4. Demands for hiding emotions	42.15 (23.81)	45.70 (24.59)	**0.096**
	5. Sensory demands	69.57 (19.09)	84.74 (18.26)	**<0.001**∗
**D2: Work organization & job content**		238.39 (70.43)	212.38 (59.61)	**<0.001**∗
	1. Influence at work	45.27 (20.50)	46.53 (21.36)	**0.496**
	2. Possibilities for development	55.80 (21.65)	73.17 (18.32)	**<0.001**∗
	3. Degree of freedom at work	40.08 (18.79)	30.49 (19.78)	**<0.001**∗
	4. Meaning of work	65.01 (19.58)	72.35 (17.52)	**<0.001**∗
	5. Commitment to the work place	55.86 (20.79)	65.32 (19.25)	**<0.001**∗
**D3: Interpersonal relations & leadership**		347.03 (99.68)	336.59 (101.72)	**0.253**
	1. Predictability	54.77 (22.41)	58.72 (20.70)	**0.038**∗
	2. Role clarity	59.61 (12.93)	59.78 (12.65)	**0.876**
	3. Role conflicts	48.84 (20.69)	50.19 (19.98)	**0.450**
	4. Quality of leadership	59.81 (26.91)	61.17 (26.90)	**0.571**
	5. Social Support	48.80 (21.22)	50.95 (22.66)	**0.270**
	6. Feedback at work	43.11 (24.67)	48.05 (25.21)	**0.027**∗
	7. Social relations	64.59 (22.09)	56.65 (25.99)	**<0.001**∗
	8. Sense of community	72.97 (20.32)	78.23 (17.96)	**0.002**∗
**D4:Work-individual interface**		97.28 (42.95)	105.46 (40.68)	**0.029**∗
	1. Insecurity at work	50.13 (35.05)	62.76 (32.82)	**<0.001**∗
	2. Job satisfaction	53.00 (19.91)	57.11 (19.54)	**0.020**∗
**D5: Health and well-being (individual)**		220.50 (92.10)	181.69 (97.35)	**<0.001**∗
	1. General health	65.11 (18.80)	73.73 (16.73)	**<0.001**∗
	2.Mental health	60.61 (19.31)	64.07 (21.75)	**0.059**
	3.Vitality	58.27 (21.19)	65.05 (22.07)	**0.001**∗
	4.Behavioural stress	34.49 (24.86)	29.00 (24.24)	**0.013**∗
	5. Somatic stress	39.29 (16.67)	39.17 (21.32)	**0.944**
	6. Cognitive stress	31.67 (20.73)	21.58 (22.06)	**<0.001**∗

∗ Statistically significant relationship (p < 0.05).

In multivariate analysis, adjustments were done to confounding factors, and with the exception of work-individual interface, significant differences were still observed in the three remaining domains (Table 3).

**Table 3:** Multiple regression modeling of the association between psychosocial domains and other variables.

Dependent Variable	Job Demand		Job Content		Interpersonal relations & leadership		Work-individual interface		Health & Well being	
	B	Sig	B	Sig	B	Sig	B	Sig	B	Sig
**Variables in model: (Constant)**	331.31	<0.001∗	335.97	<0.001∗	437.17	<0.001∗	140.64	<0.001∗	187.90	0.001∗
**Job type (Operational or office work)**	25.07	0.001∗	−27.76	<0.001∗	−6.39	0.58	−0.49	0.91	−28.28	0.01∗
**Age**	−2.38	<0.001∗	−0.68	0.30	−0.69	0.51	−1.32	0.001∗	−2.29	0.02∗
**Gender**	−6.30	0.52	23.25	0.02∗	15.26	0.32	−5.20	0.39	12.87	0.37
**Marital status**	−8.08	0.30	−26.08	0.001∗	−30.57	0.01∗	−1.97	0.68	−6.46	0.57
**Education status**	−6.08	0.10	−2.10	0.55	5.67	0.32	0.19	0.93	17.98	0.001∗
**Type of employment**	−12.89	0.22	8.12	0.43	−11.42	0.48	19.26	0.003∗	30.04	0.05
**Working years**	2.21	0.007∗	.55	0.48	0.06	0.96	0.98	0.05	3.03	0.01∗
**Working Hours per week**	1.03	0.01∗	-.32	0.41	0.18	0.78	0.09	0.72	0.37	0.53
**Directorship status**	7.24	0.43	−23.73	0.009∗	−22.40	0.13	−13.9	0.01∗	−15.02	0.27
**Salary**	−4.85	0.43	−12.01	0.04∗	−17.35	0.08	−9.86	0.01∗	−9.94	0.28
**Smoking status**	5.47	0.62	6.50	0.54	16.40	0.34	−1.6	0.81	47.39	0.004∗
**Night Shift status**	4.44	0.55	12.25	0.09	1.26	0.91	2.96	0.51	19.65	0.07
**Model R square**	0.14		0.16		0.06		0.18		0.12	

∗ Statistically significant relationship (p < 0.05).

In the "job demand" domain, operational group scored higher (worse), with differences in cognitive and sensory demands scales. Yet, in these scales, both groups were in the red area (scores more than 60). In the “job content” domain, the operational group scored higher in "possibility for development", "meaning of work", and "commitment to the workplace" scales. However, the administrative group had higher scores in "degree of freedom at work" scale and had better state.

In the "inter personal relationship" domain, no statistically significant difference was observed between the two groups. The operational group was in a better state in "feedback at work" and "sense of community" scales, but worse in "social relations". In "work-individual interface" domain, multivariate analysis showed no significant difference between the two groups, and the operational personnel had higher score in "insecurity at work", but still in a better state in terms of job satisfaction. In the health and wellbeing domain, the operational personnel scored lower (better) and had better general health and lower behavioral-cognitive stress; yet, still, in this domain, both groups were generally in the green area (scores more than 60 for the scales related to health and less than 40 for the scales related to stress).

## 4 Discussion

In this study, psychosocial factors in one electricity distribution company were assessed using Persian standard version of COPSOQ and compared between office and operational workers in terms of different domains and scales of this questionnaire. We detected a satisfactory discrimination between white and blue-collar workers in some scales of the questionnaire. Some other factors assessed by COPSOQ are general and we did not expect to differ between occupational groups.

There were differences between these two groups in terms of physical work conditions, including greater exposure of the operational group to electromagnetic waves, cold, heat, and sunlight. They were also different in terms of ergonomic conditions [37]. Exposure to physical and ergonomic hazards may affect the workers conception from their job and illustrate a presumable source for psychosocial stress.

The operational employees showed higher occupational demands, especially in sensory and cognitive scales, which agreed with the results obtained in a study in Portugal (on a small sample size and using the short version of COPSOQ) [38]. In a domestic study conducted on professional drivers in 2014, occupational demand was high in sensory and cognitive scales [33]. The high level of occupational demand in operational electricity employees indicates high job sensitivity of employees and sensory and subjective needs including good eyesight and high concentration.

One study conducted in European steel industry concluded that COPSOQ could reliably distinguish psychosocial factors in different working groups. Their study revealed that average scores of some scales such as predictability, role conflict, social relation and influence at work exceed the reference values in blue collar workers, in contrast average scores of possibility for development, meaning at work and role clarity showed slight deviation from reference values. In their study office workers had higher score in quantitative and emotional demand scales, compared to operational group and this finding is not in consistent with results of current study [31].

The operational personnel had higher stressor in "degree of freedom at work", which meant little freedom to take a leave during working hours. The worse social relations or social interactions among the operational employees are due to the nature of their job that makes them work outside with little opportunity to interact with colleagues. The same results were obtained from the study conducted in European steel industry [31]. However, the operational personnel were in a better state in "possibilities for development" scale, which may be due to greater diversity of work, initiative, and greater use of expertise and opportunity to learn new things, comparing to office workers. They were also better in "meaning of work" scale, which indicates greater sense of purpose, attachment of importance to the job, and motivation in this group. The operational group's high score in "commitment to the workplace" shows their greater commitment to their workplace, and despite their lower social interaction and possibility to talk to their colleagues, they showed greater "sense of community" or work sociability, and better job relations, which can help them in sensitive tasks.

In one study conducted on Malaysian railway workers and compared job stress between blue and white-collar workers by using the Perceived Stress Scale-10 questionnaire, white-collar employees had higher perceived stress. [39].

In univariate analysis, the operational employees scored poorly in the “work-individual interface” domain. However, regression analysis and adjustments for confounding factors showed other various factors affecting the difference in this domain (insecurity at work and job satisfaction scales) rather than job type (office or operational), including type of employment (B = 19.26), directorship status (B = 13.9), and income (B = 9.86).

There was a relatively higher feeling of health among the operational group, which might be attributed to recruitment of healthier personnel due to the nature of the job, and transfer of unhealthy personnel to office sections. According to regression analysis on the dependent variable of health, the biggest impact on feeling of health was due to smoking status (B = 47.39), job type (B = 25.9), and education level of the employees (B = 17.98), respectively.

### 4.1 Study limitations

There are very few objective tools for measuring psychosocial factors, and thus, subjective measurement of these factors was among study limitations. Exposure to physical and ergonomic hazards can affect the workers conception and satisfaction from their job and these factors were not measured and compared in this study, but we believe that comprehensive assessment of psychosocial hazards in work place is a strong point of current study. Besides, the effect of a healthy worker may affect the difference between the two groups, which may have been due to employment of healthier personnel in the operational section. This study was conducted in one electricity distribution company in Iran, and generalization of results to other companies may not be appropriate. Future studies by recruiting participants from different companies over the country can provide higher external validity.

### 4.2 Conclusion

This study can indicate the significance of psychosocial hazards in electricity distribution industry. Transferring this knowledge to policy makers of this industry is helpful for choosing better strategies to cope with these hazards and decreasing the hidden costs caused by adverse outcomes of them.

In this study, significant differences were found between office and operational employees in workplace psychosocial factors in the electricity distribution industry. Knowledge of such differences may lead to different approaches in occupational and organizational planning and thus help with greater productivity. Further studies notably cohort type with greater sample size are warranted for better evaluation of psychosocial work environments in different job groups after controlling other exposures.

## Declarations

### Author contribution statement

O. Aminian: Conceived and designed the experiments; Wrote the paper.

A. Moradi: performed the experiments; Contributed reagents, materials, analysis tools or data; Wrote the paper.

S. Eftekhari: Conceived and designed the experiments; Analyzed and interpreted the data; Wrote the paper.

### Funding statement

This research did not receive any specific grant from funding agencies in the public, commercial, or not-for-profit sectors.

### Competing interest statement

The authors declare no conflict of interest.

### Additional information

No additional information is available for this paper.
